# A linkage map for the B-genome of *Arachis *(Fabaceae) and its synteny to the A-genome

**DOI:** 10.1186/1471-2229-9-40

**Published:** 2009-04-07

**Authors:** Márcio C Moretzsohn, Andrea VG Barbosa, Dione MT Alves-Freitas, Cristiane Teixeira, Soraya CM Leal-Bertioli, Patrícia M Guimarães, Rinaldo W Pereira, Catalina R Lopes, Marcelo M Cavallari, José FM Valls, David J Bertioli, Marcos A Gimenes

**Affiliations:** 1 Embrapa Recursos Genéticos e Biotecnologia, C.P. 02372, CEP 70.770-900, Brasília, DF, Brazil; 2 Departamento de Genética, IB-UNESP, Rubião Jr, CEP 18618-000, Botucatu, SP, Brazil; 3 Universidade Católica de Brasília, Campus II, SGAN 916, CEP 70.790-160, Brasília, DF, Brazil

## Abstract

**Background:**

*Arachis hypogaea *(peanut) is an important crop worldwide, being mostly used for edible oil production, direct consumption and animal feed. Cultivated peanut is an allotetraploid species with two different genome components, A and B. Genetic linkage maps can greatly assist molecular breeding and genomic studies. However, the development of linkage maps for *A. hypogaea *is difficult because it has very low levels of polymorphism. This can be overcome by the utilization of wild species of *Arachis*, which present the A- and B-genomes in the diploid state, and show high levels of genetic variability.

**Results:**

In this work, we constructed a B-genome linkage map, which will complement the previously published map for the A-genome of *Arachis*, and produced an entire framework for the tetraploid genome. This map is based on an F_2 _population of 93 individuals obtained from the cross between the diploid *A. ipaënsis *(K30076) and the closely related *A. magna *(K30097), the former species being the most probable B genome donor to cultivated peanut. In spite of being classified as different species, the parents showed high crossability and relatively low polymorphism (22.3%), compared to other interspecific crosses. The map has 10 linkage groups, with 149 loci spanning a total map distance of 1,294 cM. The microsatellite markers utilized, developed for other *Arachis *species, showed high transferability (81.7%). Segregation distortion was 21.5%. This B-genome map was compared to the A-genome map using 51 common markers, revealing a high degree of synteny between both genomes.

**Conclusion:**

The development of genetic maps for *Arachis *diploid wild species with A- and B-genomes effectively provides a genetic map for the tetraploid cultivated peanut in two separate diploid components and is a significant advance towards the construction of a transferable reference map for *Arachis*. Additionally, we were able to identify affinities of some *Arachis *linkage groups with *Medicago truncatula*, which will allow the transfer of information from the nearly-complete genome sequences of this model legume to the peanut crop.

## Background

Peanut (*Arachis hypogaea *L.) is one of the most important crops in tropical and subtropical regions of the world. Peanut is used as both human and animal food, being a valuable source of protein and oil [1,2]. The genus *Arachis *(Leguminosae or Fabaceae) is native to South America and contains 80 described species assembled into nine taxonomical sections, according to their morphology, geographic distribution and sexual compatibility [3,4]. The *Arachis *section includes the species that can be crossed to *A. hypogaea *and encompasses 29 diploid species and the tetraploid species *A. hypogaea *and *A. monticola *[3,4].

Cultivated peanut is an allotetraploid (2n = 4× = 40 chromosomes) with two genome types, A and B, which are found separately in the wild species of the *Arachis *section. The A-genome species are diploids characterized by the presence of a so-called A chromosome pair [5], of reduced size and with a lower level of euchromatin condensation in comparison to the other chromosomes [6]. Diploid species of the section *Arachis *with 2n = 20 and lacking the A chromosome pair are usually considered to share the B-type genome, although they are much more heterogeneous and may present variant forms of this B-genome. One species, *A. glandulifera*, revealed very poor homologies with all A and B genome taxa, and is considered to have a D genome [7,8]. Three other species show 2n = 18 chromosomes [9-11] and their genomic affinities are not clear.

*Arachis hypogaea *was originated via hybridization of two diploid wild species, probably *A. duranensis *(A-genome) and *A. ipaënsis *(B-genome), followed by a rare spontaneous duplication of chromosomes [6,12-14]. The resulting tetraploid plant would have been reproductively isolated from its wild diploid relatives. This isolation, coupled with the origin through a probably single hybridization event [13,15-17], leads to a limited genetic diversity of peanut, as observed in different studies using molecular markers [13,15-17]. In contrast, wild diploid *Arachis *species are genetically more diverse [18-20], providing a rich source of variation for agronomical traits, and DNA polymorphisms for genetic and genomic studies [21-23].

As a consequence, most of the linkage maps developed for *Arachis *included wild species as progenitors, the exception being the *A. hypogaea *map that has been recently published [24]. These maps are based on RFLP [25,26], RAPD [27], and more recently, microsatellite markers [24,28]. In this latter study [28] we used a diploid population from a cross between *A. duranensis *and the closely related *A. stenosperma*, both having A-type genomes, the former being the most probable A genome donor to cultivated peanut. This map, which essentially provides genetic information for half the genetic component of *A. hypogaea*, has more recently been updated with new microsatellites, RGAs, AFLPs, and single-copy gene-based markers (anchor markers) (unpublished data).

Microsatellite markers are the ideal markers for the development of linkage maps, as they are multiallelic, highly polymorphic, typically co-dominant, and PCR-based markers. Additionally, they can often be transferred between different populations and even related species [28-31]. Therefore different maps constructed with common microsatellite markers can be aligned, allowing information from the different maps to be accumulated, helping to confirm linkage orders and providing information on the genome evolution of related species.

The aim of this study was to create a linkage map for the *Arachis *B-genome to complement the previously published A-genome map and effectively to provide a linkage map for tetraploid peanut in two separate diploid components. For that, we made an F_2 _population from a cross between the most probable B-genome donor of cultivated peanut, *A. ipaënsis *[13,14], and the very closely related *A. magna*. In order to facilitate map comparisons we used the same set of microsatellite markers used for the construction of the A-genome map, with the addition of some recently published markers, 75 newly developed microsatellite, 19 EST-STS markers and 11 strategically chosen anchor markers, which are single copy genic markers that are ideal for the alignment of genomes [32-34].

## Results

### Interspecific hybridization

Several crossings between *A. ipaënsis *and *A. magna *were made. Seven plants of *A. ipaënsis *(K30076) and six of *A. magna *(K30097) were used as female parents (see Additional file 1). A total of 993 flowers were cross-pollinated, of which 515 and 478 had *A. ipaënsis *and *A. magna *as female parents, respectively. A total of 556 viable seeds were obtained, being 313 (56%) from *A. ipaënsis *× *A. magna *crosses and 243 (44%) from *A*. *magna *× *A. ipaënsis *crosses. Hybrids were identified using the SSR marker Ah-282 visualized in 3% agarose gels. The number of seeds obtained from the 23 self-pollinated F_1 _individuals was high, ranging from 50 to 165, with an average of 92. The F_1 _plant obtained from cross 4 (see Additional file 1), which produced the highest number of seeds (165) was selected to generate the F_2 _mapping population.

### Marker development and analysis

#### Genomic microsatellites

Forty primer pairs were developed using the three genomic libraries enriched for AC/TG and AG/TC repeats (see Additional file 2) and were screened against the progenitors of the mapping population. Repeats were, as expected, almost entirely composed of dinucleotides (Table 1). Nine out of the 40 primer pairs (22.5%) were polymorphic, including one dominant marker (present in *A. ipaënsis *and absent in *A. magna*); seven (17.5%) were monomorphic; 13 (32.5%) did not amplify any fragment, and 11 (27.5%) did not allow precise analyses (Table 2).

**Table 1:** Characteristics of the newly developed markers

**Repeat motif**	**Genomic SSR**	**EST-SSR**
Dinucleotides	38 (95.0)	17 (48.6)
Trinucleotides	-	13 (37.1)
Tetranucleotides	-	1 (2.9)
Di- and trinucleotides	1 (2.5)	4 (11.4)
Di- and tetranucleotides	1 (2.5)	-

Total	40	35

Number of the newly developed EST- and genomic SSR markers detected per repeat size class. Numbers in parentheses refer to the percentages of the total.

**Table 2:** Polymorphism levels detected for the different markers.

	**Genomic SSR**	**EST-SSR**	**EST-STS**
**New markers**			
Polymorphic	9 (22.5%)	9 (25.7%)	2 (10.5%)
Monomorphic	7 (17.5%)	15 (42.9%)	10 (52.6%)
No amplification	13 (32.5%)	5 (14.3%)	1 (5.3%)
Poor amplification	11 (27.5%)	6 (17.1%)	6 (31.6%)
Total	40	35	19
			
**All markers**			
Polymorphic	123 (22.1%)	43 (22.8%)	2 (10.5%)
Monomorphic	267 (48.0%)	106 (56.1%)	10 (52.6%)
No amplification	119 (21.4%)	17 (9.0%)	1 (5.3%)
Poor amplification	47 (8.5%)	23 (12.1%)	6 (31.6%)
Total	556	189	19

Summary of the results obtained for the three types of markers detected after screening against the two BB genome species (*A. ipaënsis*, accession K30076 and *A. magna*, accession K30097) used as progenitors of the F_2 _mapping population.

A total of 556 genomic SSR markers (the 40 developed here plus 516 cited in literature) were tested against *A. ipaënsis *(K30076) and *A. magna *(K30097). Of these, 123 (22.1%) were polymorphic (including one dominant marker); 267 (48.0%) were monomorphic, and 166 (29.9%) did not amplify any interpretable fragment (Table 2).

#### EST-SSR markers

Out of the 738 unique sequences obtained from the two *A. hypogaea *cDNA libraries enriched for expressed genes in response to *Cercosporidium personatum *[35], 61 (8.3%) presented SSRs with more than five repeats and 35 primer pairs could be designed (see Additional file 2). Frequencies of the SSR repeat types are shown in Table 1. Di- and trinucleotides were the most abundant repeats. Out of the 35 primer pairs screened against both progenitors, nine (25.7%) were polymorphic, 15 (42.9%) were monomorphic, six (17.1%) did not produced any amplification, and five (14.3%) resulted in low intensity or multiple-band patterns, and were excluded from the analyses (Table 2). The homologies between the sequences and genes are shown in Additional file 2.

Of the 189 EST-SSR markers screened against *A. ipaënsis *and *A. magna *(35 new plus 154 already published), only 17 (9.0%) did not amplify any product. A total of 43 EST-SSR markers (22.8%) were polymorphic, 106 (56.1%) were monomorphic, and 23 (12.1%) were excluded due to poor or confusing amplification patterns (Table 2).

#### EST-STS markers

Nineteen primer pairs were designed from ESTs with homologies to plant genes involved in defense processes against biotic stress (see Additional file 2). Of these, two detected polymorphism against both progenitors, ten were monomorphic, one did not amplify any product, and six resulted in low intensity or multiple band patterns, and were excluded from the analyses (Table 2).

#### SNP markers

Ten anchor markers and one microsatellite distributed in six linkage groups of the AA map [28,36] were selected for mapping in the BB population. These selected markers were size monomorphic between the mapping parents as judged by electrophoresis in 4% polyacrylamide gel. The PCR products were sequenced and SNPs were identified for the 11 markers. In average, one SNP was identified per 200 bp, ranging from one SNP for every 42 bp to 627 bp. These markers were separated in two multiplex groups of five/six markers each and analyzed in the parents, the F_1 _hybrid and the F_2 _population.

### Genetic Mapping

**Figure 1:**
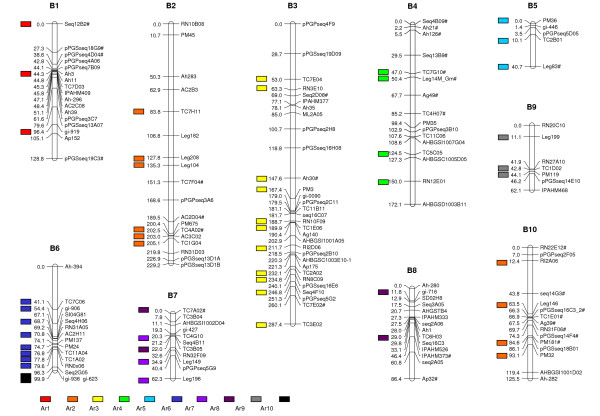
**A linkage map for the B-genome of *Arachis***. Linkage map of *Arachis *based on an F_2 _population resultant from the cross *A. ipaënsis *× *A. magna *(B-genome). The map consists of 10 linkage groups and 149 codominant markers (genomic SSR, EST-SSR, STS, and SNPs). Distorted markers (*P *< 0.05) are identified by # after the loci names. Numbers on the left of each group are Kosambi map distances. Syntenic markers between the B- and A-genome maps [28,36] are indicated by colored blocks. Colors were assigned to the A-genome linkage groups so that syntenic LG are represented by corresponding colors.

A total of 745 SSR markers were evaluated, of which 166 (22.3%) were polymorphic between the parents. Using a minimum LOD score of 3.0 and a maximum recombination fraction of 0.35, 149 markers mapped into 10 linkage groups. These markers included 106 genomic SSRs, 32 EST-SSRs, two EST-STS, and nine anchor markers. The map covered a total distance of 1,294.4 cM (Figure 1). Groups ranged from 40.7 cM (5 markers) to 287.4 cM (31 markers), with an average distance of 8.7 cM between adjacent markers. Linkage groups were numbered according to the LG numbers of the AA genome map [28,36] by the identification of syntenic markers. Two SSR primer pairs amplified consistently two loci (RN9A05 and pPGSseq16C3) and these markers were identified by the numbers _1 and _2 after the marker names (Figure 1). Thirty-two markers (21.5%) out of the 149 mapped markers showed deviation from the expected 1:2:1 ratio, being 24 at *P *< 0.05 and eight at *P *< 0.01. Of these, 12 markers were skewed towards *A. magna*, three markers towards *A. ipaënsis*, and 17 towards the heterozygote. Linkage groups B2 and B10 had all distorted markers with an excess of *A. magna *alleles, while LGs B1, B4, and B7 had all distorted markers skewed towards the heterozygote. The three markers with an excess of *A. ipaënsis *alleles grouped on LGs B3, B5 and B8 that also had markers with an excess of *A. magna *alleles and towards the heterozygote. Distorted markers at *P *< 0.05 were identified by # (Figure 1). Groups B6 and B9 had no distorted markers.

### Synteny analysis

A total of 51 common markers mapped in the AA and BB genome diploid maps spanned the 10 linkage groups of both maps (Figure 1). Seven LGs of the BB map (B1, B2, B3, B4, B5, B8, and B9) showed direct correspondences with seven groups of the AA map. Of these, five had all common markers mapped in the same order. From two (LG B8) to 11 (LG B3) collinear loci were identified per linkage group. The groups B2 and B10 showed common loci to group A2, and two segmental inversions were apparent (see Additional file 3). Group B2 was syntenic to the upper region of LG A2 with five collinear loci, and the group B10 in the lower region. Inversions were also detected in the LGs B1/A1 and B6/A6. Linkage groups B6 and B7 showed split syntenic relationships, with common markers mapping in two LG of the AA map, B6 with A6 and A10, and B7 with A7 and A8.

## Discussion

This linkage map was obtained using an F_2 _population derived from a cross between *A. ipaënsis *and *A. magna*. Several lines of evidence indicate that *A. ipaënsis *is the most probable donor of the B-genome to *A. hypogaea *[6,13,14,37,38]. *Arachis magna *is also a B-genome species closely related to *A. ipaënsis*, as indicated by crossability data [3], high rates of pollen viability in hybrids [39], and molecular marker analyses [17,19,20,40]. The high fertility of the crosses and low polymorphism levels between the species (22.3% of SSR markers) observed here support this close relationship, and indeed even suggest that the two names could actually correspond to a single biological species. Further studies should be carried out to check this hypothesis, as it might have important implications for the incorporation of new wild alleles in cultivated peanut: so far there are many collected accessions of *A. magna *and only one available accession of *A. ipaënsis*. However, regardless the taxonomic status of the species, it is clear that both genomes used to construct the map are similar to the B-genome of *A*. *hypogaea *and that the linkage map is probably a good representation of it.

The DNA polymorphism within this population is lower than the populations used for the construction of previously published *Arachis *maps: 51% for RFLP probes in the *A. stenosperma *× *A. cardenasii *derived population [25]; 40% for RFLP probes in the *Arachis hypogaea *× synthetic amphidiploid {*A. batizocoi *× (*A. cardenasii *× *A. diogoi*)}^4× ^population [26]; and 47% for SSR markers in the *A. duranensis *× *A. stenosperma *derived population [28]. This low polymorphism has been compensated by the large number of SSR markers developed for *Arachis *over the past few years [19,20,28,40-45], which has enabled the development of this linkage map. On the other hand, the segregation distortion of 21.5% is in the same range as the distortion found in many intraspecific maps [46-48]. Linkage groups B2 and B10 had all distorted markers with an excess of *A. magna *alleles, while LG B1, B4, and B7 had all distorted markers skewed towards the heterozygote. These groupings of distorted markers suggest that some regions of the chromosome are more prone to segregation distortion, rather than the distortion being marker-specific.

All markers evaluated in this study were amplified using heterologous primers. Most of them were developed for *A. hypogaea *and *A. stenosperma*, and 74 markers were developed for species from other sections of the *Arachis *genus (50 primer pairs for *A. pintoi *of section *Caulorrhizae *and 24 for *A. glabrata *of section *Rhizomatosae*), confirming the high transferability of SSR markers within the *Arachis *genus. From 745 markers tested, 609 (81.7%) allowed the amplification of PCR products in *A. ipaënsis *and/or *A. magna*. As expected, the level of transferability varied among the different types of primers tested. Microsatellites based on expressed genic regions (EST-SSR and STSs) showed higher transferability levels (91.0% and 94.7%, respectively) than random genomic microsatellites (78.6%). This confirms previous findings that markers based on cDNA sequences are more transferable among species than random markers, such as genomic SSRs, since they are based on coding regions, which are generally more conserved that non coding regions [49-54].

The number of repeats found in the genomic microsatellite markers was, in general, higher (5 to 64 repeats) than the number in expressed genic microsatellites (5 to 16 repeats). This difference was not reflected in the polymorphism levels found for these two sources of primers: 22.8% of the EST-SSRs and 22.0% of the genomic SSRs. These findings are in agreement with our previous results for wild species and contrasts with cultivated peanut, where longer microsatellites have higher polymorphism [28].

The present map comprised 10 linkage groups, with 149 loci spanning a total map distance of 1,294.4 cM, which corresponds to the haploid chromosome number of the progenitor species n = 10 [3]. The total length obtained is similar to the sizes described for the other two co-dominant marker-based linkage maps published for diploid species of *Arachis*: 1,063 cM for an RFLP based map developed using an *A. stenosperma *× *A. cardenasii *cross [25] and 1,230.9 cM found for a microsatellite based map developed using an *A. duranensis *× *A. stenosperma *cross [28]. This size is also comparable to half of the 2,210.0 cM found for a published tetraploid map for *Arachis *spp. [26]. However, seventeen (10.2%) of the 166 segregating markers remained unlinked, suggesting that at least parts of the genome have not been covered by this map.

Twenty five percent of the mapped markers were developed from cDNA libraries (33 EST-SSR and two STS markers). Some of them had similarity to genes of known function, including genes involved in the photosynthesis process and in responses to biotic stresses. For instance, marker AHBGSD1002H08 (LG B8) showed similarity to a tissue specific gene coding for a prolin-rich protein of soybean (E-value = 3.0 × 10^-27^), that has the expression induced by salicylic acid, virus infection, circadian rhythm and salinic and drought stresses, indicating this gene may have an important role in the response to multiple internal and external factors [55]. Marker AHBGST1002B04 showed similarity to dihiydro-isoflavone redutase (E-value = 3.0 × 10^-57^), that is an enzyme involved in the synthesis of different flavonoids, and some of them, such as flavones and the 3-deoxyanthocyanidina, are involved in the plant defense process [56]. Linkage maps that contain genic markers can facilitate the finding of genes of interest, as ESTs mapping in regions with QTLs are good candidates to be involved in the trait and being an alternative to positional cloning [47,57].

A total of 42 microsatellite markers in common with the A-genome map [28] were placed on this B-genome map. In order to increase the number of shared markers, nine anchor markers [32-34] selected from the A-map [36] were placed on the B-map using SNPs. The comparison of the 51 shared markers revealed associations between maps and apparently high levels of synteny, since all but one of the B linkage groups show single main correspondences to the A-map. This seems largely consistent with the observed for homeologous groups in the published tetraploid map of *Arachis *[26] with perhaps the main differences being: in the tetraploid study, one large B linkage group shows no marker correspondences to the A genome, whilst in this study no "orphan" linkage groups are present; and in this study two B linkage groups correspond to one A (B2 and B10 to A2), a situation not observed in the tetraploid map.

**Figure 2:**
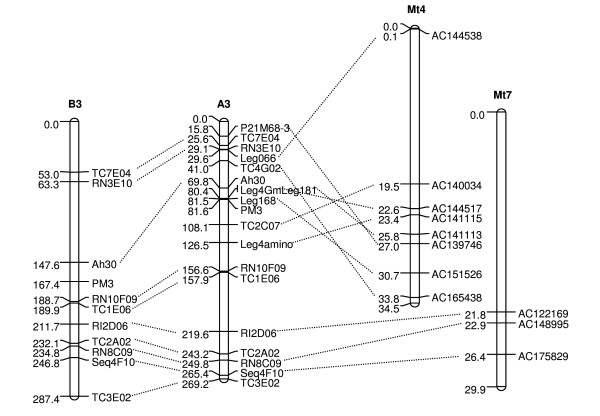
**An example of synteny between A- and B- genomes of *Arachis *and *Medicago***. Alignment of linkage group B3 of the developed map with the A-genome (LG A3) and *Medicago truncatula *(LG Mt4 and Mt7).

The integration of the A- and B-genome *Arachis *maps effectively increases the information content of both maps. The A-genome map contains candidate genes and QTLs for disease resistance, and has been aligned with the genomes of the model legumes *Lotus *and *Medicago *and with the bean genetic map [36,58]. Much of this information is likely to be transferable to the B-map. As an example, Figure 2 shows an alignment of the B-map through the A-map with *Lotus*, whose genome sequence was recently published [59]. This type of alignment allows the inference of the position of candidate genes from a whole genome sequence on the B-genome map.

## Conclusion

Here we present a microsatellite-based map for the B-genome of *Arachis *and its integration with an A-genome map. The development of these maps, based on markers that are highly transferable and simple to use will facilitate the identification and introgression of useful genes from both A-type and B-type wild genomes into cultivated peanut. These maps will also be used as reference for future cultivated peanut maps and for the development of introgression lines which are underway. Both the B-genome population described here and the A-genome population [28], have now been developed into F_5 _RIL (Recombinant Inbred Lines) populations which will facilitate the even broader use of these map and marker resources.

## Methods

### Plant material

The F_2 _population composed of 93 plants was obtained by selfing a unique F_1 _plant derived from a cross between *A. ipaënsis *(accession K30076), used as the female parent, and *A. magna *(K30097), used as the male. Accession K30097 is the holotype of *A. magna*, while K30076 originate from the same collection site of the type specimen of *A. ipaënsis *[3,4]. Plants were obtained from the Brazilian *Arachis *germplasm collection, maintained at Embrapa Genetic Resources and Biotechnology – CENARGEN (Brasília-DF, Brazil).

### DNA extraction

Total genomic DNA was extracted from young leaflets essentially as described by Grattapaglia & Sederoff (1994) [60]. The quality and quantity of the DNA were evaluated in 1% agarose gel electrophoresis and spectrophotometer (Genesys 4 – Spectronic).

### Marker development and analysis

The same set of microsatellite markers used in Moretzsohn et al., 2005 [28] was used for screening for polymorphism between the parents. In addition, some markers recently published [44,45] were used, as well as the newly developed one, as follows:

#### Development of genomic DNA libraries enriched for microsatellites

Three libraries were developed using genomic DNA isolated from leaves of *A*. *hypogaea *(section *Arachis*), *A. glabrata *(section *Rhizomatosae*) and *A. pintoi *(section *Caulorrhizae*). For each library, about nine micrograms of DNA were digested with *Sau*3AI (Amersham Biosciences, UK) and electrophoresed in 0.8% low melting agarose gels to select fragments ranging from 200 to 600 bp. The selected fragments were purified from the agarose gels using phenol/chloroform, and ligated into *Sau*3AI specific adaptors (5'-cagcctagagccgaattcacc-3' and 5'-gatcggtgaaatcggctcaggctg-3'). The ligated fragments were hybridized to biotinylated (AC)_15 _and (AG)_15 _oligonucleotides and isolated using streptavidin-coated magnetic beads (Dynabeads Streptavidin, Dynal Biotech, Norway). The eluted fragments were amplified using one adaptor-specific primer, cloned into the pGEM-T Easy vector (Promega, WI, USA) and transformed into DH5α *E. coli *cells with blue/white selection (Invitrogen, CA, USA). Plasmid DNAs of the positive clones were isolated using the 'CONCERT Rapid Plasmid Purification Miniprep System', as described by the manufacturer (Invitrogen, CA, USA) and sequenced with an ABI Prism 377 automated sequencer using the 'BigDye Terminator Cycle Sequencing Kit', version 3.1 (Applied Biosystems, CA, USA).

#### EST-SSR and EST-STS marker development

EST-SSRs were developed from 883 EST sequences obtained from a recently constructed Suppression Subtractive Hybridization – SSH library of *A. hypogaea *enriched for expressed genes in response to *Cercosporidium personatum *[35] using the software described below. In addition, 14 *A. hypogaea *ESTs were selected due to their similarity to genes involved in defense mechanisms, identified using BlastX analyses [61]. From these, 12 sequences had no SSR repeats, but were used for primer design to develop STS (Sequence tagged sites) markers. Primers were also designed for an EST of unknown function (AHBGSI1002C10), for a sequence similar to a dienelactone hydrolase family protein of *Arabidopsis thaliana *(AHBGSI1006D06) and for three ESTs of putative intron adjacent sequences (AHBSI1001D05-I1, AHBSI1002C11-I1 and AHBSI1009D07-I2) that were selected using an unpublished software developed by Dr. Wellington Martins, Universidade Católica de Goiás, Brazil.

#### Primer design

Sequences were processed and assembled by using the Staden package [62] with the repeat sequence finding module TROLL [63] and Primer3 [64]. Sequences with more than five motif repeats were chosen for primer design. The parameters for primer design were: (1) primer size ranging from 18 bp to 25 bp with an optimal length of 20 bp; (2) primer *T*_m _(melting temperature) ranging from 57°C to 63°C with an optimal temperature of 60°C; and (3) GC content ranging from 40% to 60%. Default values were used for the other parameters.

#### PCR amplifications

PCR reactions contained 5 ng of genomic DNA, 1 U of *Taq *DNA polymerase (Amersham Biosciences), 1× PCR buffer (200 mM Tris pH 8.4, 500 mM KCl), 1.5–2.0 mM MgCl_2_, 200 μM of each dNTP, and 0.4 μM of each primer, in a final reaction volume of 10 μl. Amplifications were carried out in a PTC100 thermocycler (MJ Research Inc., MA, USA). PCR conditions were: 96°C for 5 min, followed by 32 cycles of 96°C for 30 s, 48–62°C (annealing temperature depending on primer pair, see Additional file 2) for 45 s, 72°C for 1 min, with a final extension for 10 min at 72°C. PCR products were separated by electrophoresis on denaturing polyacrylamide gels (6% acrylamide:bisacrylamide 29:1, 5 M urea in TBE pH 8.3), stained with silver nitrate [65]. Some SSR markers highly contrasting between the progenitors of the mapping population were run on 3% agarose Metaphor (FMC Bioproducts, PA, USA) gels stained with ethidium bromide.

### SNPs identification and analysis

Ten anchor markers and one microsatellite distributed in six linkage groups of the AA map [28,36] were selected for mapping in the BB population. Markers from A-genome linkage groups that had few markers in common with an initial version of the B-map were preferentially chosen. The identification of SNPs and single base extension (SNaPshot) analysis was performed essentially as described by Alves et al. (2008) [66]. Primers were designed using the program Primo SNP 3.4, available at  (Chang Bioscience). The SNP in the consensus sequence of both progenitors was replaced by a degenerated IUPAC code for primer design. Non-homologous polynucleotides (dGACT)_n _were added to the 5'-end of each primer to enable the analysis in multiplexes (see Additional file 2), using the commercial system ABI PRISM^® ^SNaPshot™ Multiplex Kit (Applied Biosystems). Absence of hairpins and self-complementarity of all SNP primers were checked by the software Autodimer [67].

### Map construction

A total of 745 SSR, 19 STS and 11 SNP markers were screened against the two progenitors of the mapping population. These included the 105 newly developed markers (see Additional file 2) plus another 670 published microsatellite markers [19,20,28,40-45,68-70]. Polymorphic markers were analyzed on the mapping population consisting of 93 F_2 _individuals. A χ^2 ^test was performed to test the null hypothesis of 1:2:1 segregation on all scored markers. The linkage analysis was done using Mapmaker Macintosh version 2.0 [71]. A minimum LOD score of 4.0 and maximum recombination fraction (θ) of 0.35 were set as thresholds for linkage groups determination with the "group" command. The most likely marker order within each LG was estimated by the matrix correlation method using the "first order" command. Marker orders were confirmed by comparing the log-likelihood of the possible orders using multipoint analysis ("compare" command) and by permuting all adjacent triple orders ("ripple" command). After establishment of the group orders, the LOD score was set to 3.0 in order to include additional markers in the groups. The "try" command was then used to determine the exact position of the new markers within each group. The new marker orders were again confirmed with the "first order", "compare", and/or "ripple" commands. Recombination fractions were converted into map distances in centimorgans (cM) using the Kosambi's mapping function.

## Authors' contributions

All authors read and approved the final manuscript. MCM carried out the analysis for genetic map construction, participated in the synteny analysis and drafted the manuscript. AVGB carried out the mapping population construction, participated in the development and analysis of SSR and STS markers and drafting the manuscript. DMTAF carried out the identification and analysis of SNP markers. CT and MMC participated in SSR and STS markers analysis. SCMLB and PMG participated in the SSR and synteny analyses. RWP coordinated the identification and analysis of SNP markers. CRL participated in conceiving the study. JV participated in the conception of the project and provided the germplasm. DJB participated in SSR, STS and SNP development and analysis, carried out the synteny analysis and participated in drafting the manuscript. MAG participated in conceiving the study, coordinated the SSR and STS markers development and analysis, and participated in drafting the manuscript.

## Supplementary Material

Additional File 1 **Data of crossings between *A. ipaënsis *(accession K30076) and *A. magna *(K30097)**. The data provides the number of viable seeds obtained by crossing *A. ipaënsis *(accession K30076) and *A. magna *(K30097) and by selfing F_1 _hybrid individuals. Click here for file

Additional File 2 **Features of the newly developed markers**. The data provides the details of the new set of markers, being 40 genomic SSR, 35 EST-SSR, 19 STS, and 11 SNPs. Click here for file

Additional File 3 **Relationships between the 10 linkage groups of the A- and B-genome maps**. The data provides the affinities between the A- and B-genome linkage maps of *Arachis*. Click here for file
