# Supplementary material for: A linkage map for the B-genome of Arachis (Fabaceae) and its synteny to the A-genome
Source: BMC Plant Biol. 2009 Apr 7;9:40. doi: 10.1186/1471-2229-9-40 (PMC2674605; doi:10.1186/1471-2229-9-40)
Supplement: Additional File 1 — Data of crossings between A. ipaënsis (accession K30076) and A. magna (K30097). The data provides the number of viable seeds obtained by crossing A. ipaënsis (accession K30076) and A. magna (K30097) and by selfing F1 hybrid individuals. [file 1471-2229-9-40-S1.doc]

**Table 1** – Crossability between *Arachis ipaënsis* (accession K30076) and *A. magna* (accession K30097).

| Cross | Female parent | Male parent | Number of flowers pollinated | Number of viable seeds |
| --- | --- | --- | --- | --- |
| 1 | *A. ipaënsis -* plant 01 | *A. magna* – plant 23 | 83 | 73 |
| 2 | *A. ipaënsis -* plant 02 | *A. magna* – plant 20 | 70 | 51 |
| 3 | *A. ipaënsis -* plant 03 | *A. magna* – plant 18 | 101 | 53 |
| 4 | *A. ipaënsis -* plant 04 | *A. magna* – plant 21 | 79 | 31 |
| 5 | *A. ipaënsis -* plant 06 | *A. magna* – plant 22 | 53 | 24 |
| 6 | *A. ipaënsis -* plant 08 | *A. magna* – plant 19 | 38 | 58 |
| 7 | *A. ipaënsis -* plant 11 | *A. magna* – plant 23 | 91 | 23 |
| **Sub-total** |  |  | **515** | **313** |
| 8 | *A. magna* - plant 12 | *A. ipaënsis -* plant 10 | 91 | 06 |
| 9 | *A. magna* - plant 13 | *A. ipaënsis -* plant 05 | 110 | 50 |
| 10 | *A. magna* - plant 14 | *A. ipaënsis -* plant 07 | 67 | 60 |
| 11 | *A. magna* - plant 15 | *A. ipaënsis -* plant 10 | 66 | 10 |
| 12 | *A. magna* - plant 16 | *A. ipaënsis -* plant 09 | 80 | 39 |
| 13 | *A. magna* - plant 17 | *A. ipaënsis -* plant 09 | 64 | 78 |
| **Sub-total** |  |  | **478** | **243** |
| **Total** |  |  | **993** | **556** |

**Table 2** – Number of F_2_ viable seeds obtained from selfcrossing of F_1_ hybrid individuals from the female parental pot number 4.

| **F_1_ Pot 4** | **F_2_ viable seeds** |
| --- | --- |
| Individual 01 – hybrid | 165 |
| Individual 02 – hybrid | 150 |
| Individual 04 – hybrid | 160 |
| Individual 05 – hybrid | 155 |
| Individual 07 – hybrid | 140 |
| Individual 08 – hybrid | 044 |
| Individual 10 – hybrid | 101 |
| Individual 11 – hybrid | 098 |
| Individual 12 – hybrid | 098 |
| Individual 13 – hybrid | 080 |
| Individual 14 – hybrid | 080 |
| Individual 15 – hybrid | 052 |
| Individual 16 – hybrid | 076 |
| Individual 17 – hybrid | 062 |
| Individual 19 – hybrid | 063 |
| Individual 20 – hybrid | 082 |
| Individual 21 – hybrid | 096 |
| Individual 22 – hybrid | 068 |
| Individual 24 – hybrid | 054 |
| Individual 25 – hybrid | 067 |
| Individual 26 – hybrid | 080 |
| Individual 27 – hybrid | 087 |
| Individual 29 – hybrid | 050 |
| **Total** | **2,108** |
