# Supplementary material for: A linkage map for the B-genome of Arachis (Fabaceae) and its synteny to the A-genome
Source: BMC Plant Biol. 2009 Apr 7;9:40. doi: 10.1186/1471-2229-9-40 (PMC2674605; doi:10.1186/1471-2229-9-40)
Supplement: Additional File 3 — Relationships between the 10 linkage groups of the A- and B-genome maps. The data provides the affinities between the A- and B-genome linkage maps of Arachis. [file 1471-2229-9-40-S3.ppt]

## Slide 1
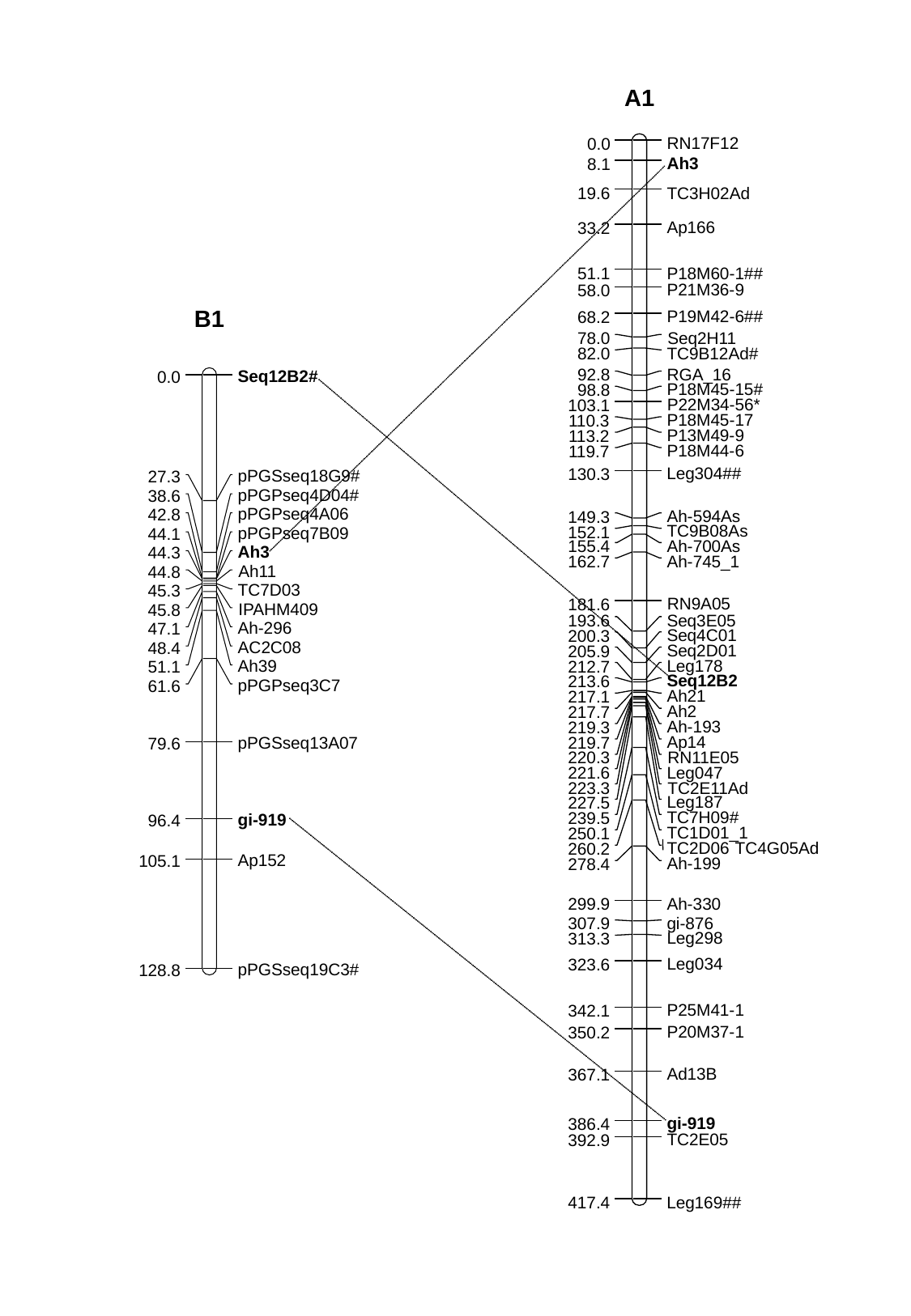

A1
RN17F12
0.0
Ah3
8.1
19.6
TC3H02Ad
Ap166
33.2
51.1
P18M60-1##
P21M36-9
58.0
P19M42-6##
68.2
78.0
Seq2H11
82.0
TC9B12Ad#
RGA_16
92.8
P18M45-15#
98.8
P22M34-56*
103.1
P18M45-17
110.3
P13M49-9
113.2
P18M44-6
119.7
Leg304##
130.3
Ah-594As
149.3
TC9B08As
152.1
155.4
Ah-700As
162.7
Ah-745_1
RN9A05
181.6
193.6
Seq3E05
Seq4C01
200.3
Seq2D01
205.9
Leg178
212.7
Seq12B2
213.6
Ah21
217.1
Ah2
217.7
Ah-193
219.3
Ap14
219.7
220.3
RN11E05
221.6
Leg047
223.3
TC2E11Ad
Leg187
227.5
TC7H09#
239.5
TC1D01_1
250.1
TC2D06
TC4G05Ad
260.2
Ah-199
278.4
299.9
Ah-330
307.9
gi-876
Leg298
313.3
Leg034
323.6
P25M41-1
342.1
P20M37-1
350.2
Ad13B
367.1
gi-919
386.4
TC2E05
392.9
417.4
Leg169##
B1
Seq12B2#
0.0
pPGSseq18G9#
27.3
pPGPseq4D04#
38.6
pPGPseq4A06
42.8
pPGPseq7B09
44.1
Ah3
44.3
Ah11
44.8
TC7D03
45.3
IPAHM409
45.8
Ah-296
47.1
AC2C08
48.4
Ah39
51.1
pPGPseq3C7
61.6
pPGSseq13A07
79.6
gi-919
96.4
Ap152
105.1
pPGSseq19C3#
128.8

## Slide 2
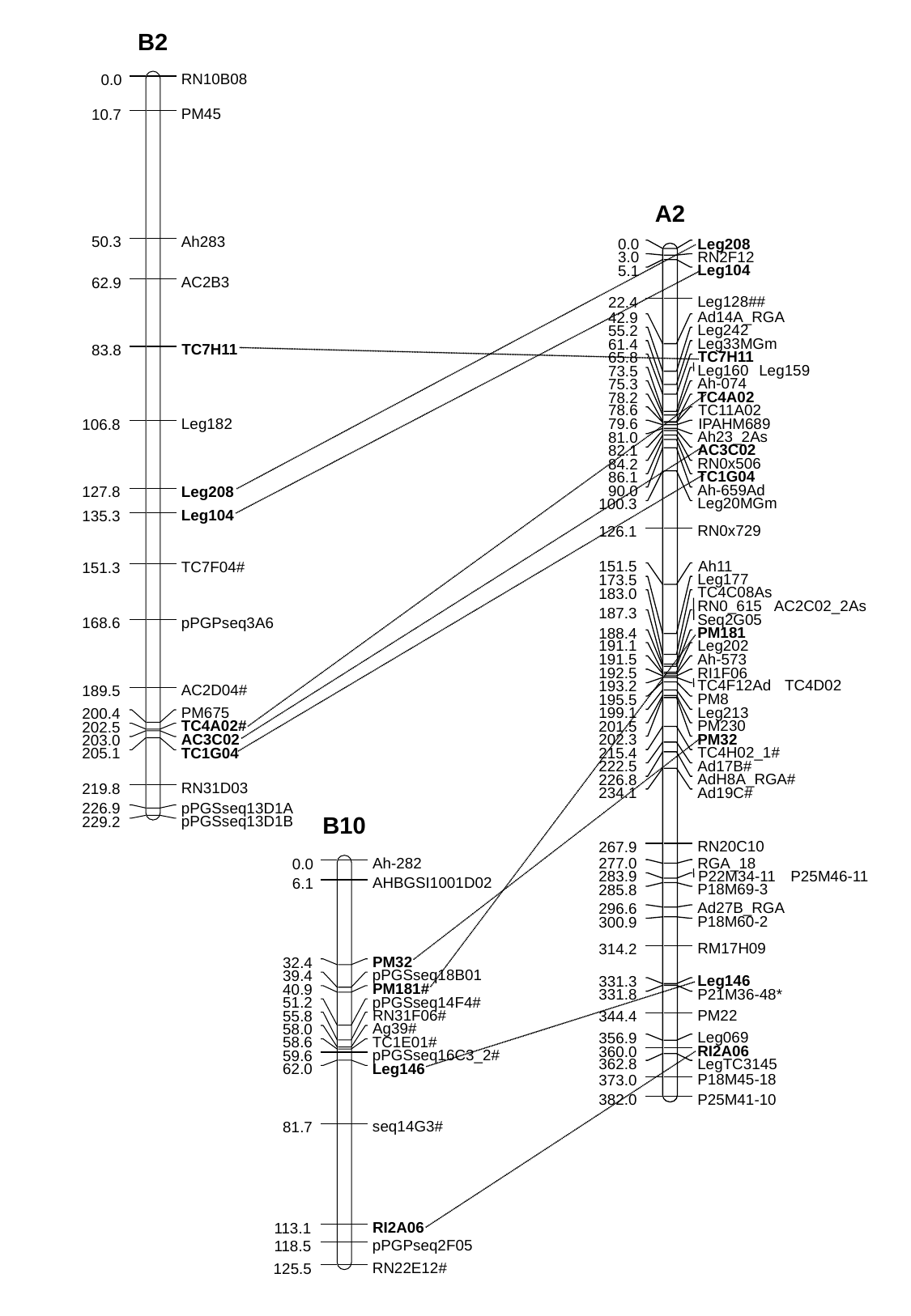

B2
RN10B08
0.0
PM45
10.7
Ah283
50.3
AC2B3
62.9
TC7H11
83.8
Leg182
106.8
Leg208
127.8
Leg104
135.3
TC7F04#
151.3
pPGPseq3A6
168.6
AC2D04#
189.5
PM675
200.4
TC4A02#
202.5
AC3C02
203.0
TC1G04
205.1
RN31D03
219.8
pPGSseq13D1A
226.9
pPGSseq13D1B
229.2
A2
0.0
Leg208
3.0
RN2F12
Leg104
5.1
Leg128##
22.4
Ad14A_RGA
42.9
Leg242
55.2
Leg33MGm
61.4
TC7H11
65.8
Leg160
Leg159
73.5
Ah-074
75.3
TC4A02
78.2
78.6
TC11A02
79.6
IPAHM689
Ah23_2As
81.0
AC3C02
82.1
RN0x506
84.2
TC1G04
86.1
Ah-659Ad
90.0
Leg20MGm
100.3
RN0x729
126.1
Ah11
151.5
Leg177
173.5
TC4C08As
183.0
RN0_615
AC2C02_2As
187.3
Seq2G05
PM181
188.4
191.1
Leg202
191.5
Ah-573
192.5
RI1F06
TC4F12Ad
TC4D02
193.2
PM8
195.5
Leg213
199.1
PM230
201.5
PM32
202.3
TC4H02_1#
215.4
Ad17B#
222.5
AdH8A_RGA#
226.8
234.1
Ad19C#
RN20C10
267.9
277.0
RGA_18
P22M34-11
P25M46-11
283.9
P18M69-3
285.8
Ad27B_RGA
296.6
P18M60-2
300.9
RM17H09
314.2
Leg146
331.3
P21M36-48*
331.8
PM22
344.4
Leg069
356.9
RI2A06
360.0
362.8
LegTC3145
P18M45-18
373.0
382.0
P25M41-10
B10
Ah-282
0.0
AHBGSI1001D02
6.1
PM32
32.4
pPGSseq18B01
39.4
PM181#
40.9
pPGSseq14F4#
51.2
RN31F06#
55.8
Ag39#
58.0
TC1E01#
58.6
pPGSseq16C3_2#
59.6
Leg146
62.0
seq14G3#
81.7
RI2A06
113.1
pPGPseq2F05
118.5
RN22E12#
125.5

## Slide 3
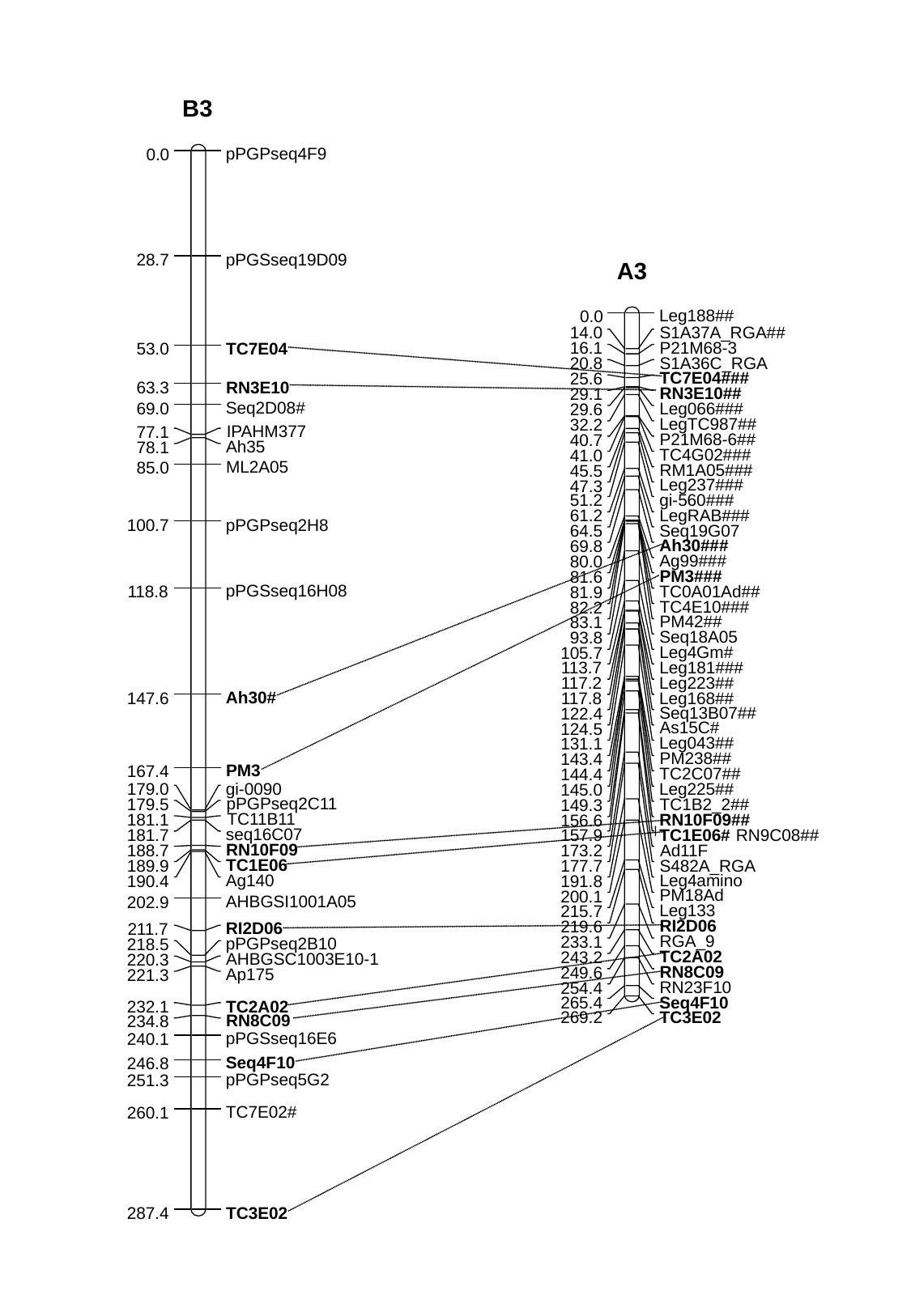

B3
pPGPseq4F9
0.0
28.7
pPGSseq19D09
53.0
TC7E04
63.3
RN3E10
Seq2D08#
69.0
IPAHM377
77.1
Ah35
78.1
ML2A05
85.0
100.7
pPGPseq2H8
pPGSseq16H08
118.8
Ah30#
147.6
PM3
167.4
179.0
gi-0090
pPGPseq2C11
179.5
TC11B11
181.1
seq16C07
181.7
RN10F09
188.7
TC1E06
189.9
Ag140
190.4
AHBGSI1001A05
202.9
RI2D06
211.7
pPGPseq2B10
218.5
AHBGSC1003E10-1
220.3
Ap175
221.3
232.1
TC2A02
RN8C09
234.8
pPGSseq16E6
240.1
Seq4F10
246.8
pPGPseq5G2
251.3
TC7E02#
260.1
287.4
TC3E02
A3
Leg188##
0.0
14.0
S1A37A_RGA##
16.1
P21M68-3
20.8
S1A36C_RGA
TC7E04###
25.6
RN3E10##
29.1
Leg066###
29.6
LegTC987##
32.2
P21M68-6##
40.7
TC4G02###
41.0
RM1A05###
45.5
Leg237###
47.3
51.2
gi-560###
61.2
LegRAB###
64.5
Seq19G07
Ah30###
69.8
Ag99###
80.0
PM3###
81.6
TC0A01Ad##
81.9
TC4E10###
82.2
PM42##
83.1
Seq18A05
93.8
Leg4Gm#
105.7
113.7
Leg181###
117.2
Leg223##
117.8
Leg168##
Seq13B07##
122.4
As15C#
124.5
Leg043##
131.1
PM238##
143.4
TC2C07##
144.4
Leg225##
145.0
TC1B2_2##
149.3
RN10F09##
156.6
157.9
TC1E06#
RN9C08##
173.2
Ad11F
177.7
S482A_RGA
Leg4amino
191.8
PM18Ad
200.1
Leg133
215.7
RI2D06
219.6
RGA_9
233.1
TC2A02
243.2
RN8C09
249.6
RN23F10
254.4
265.4
Seq4F10
269.2
TC3E02

## Slide 4
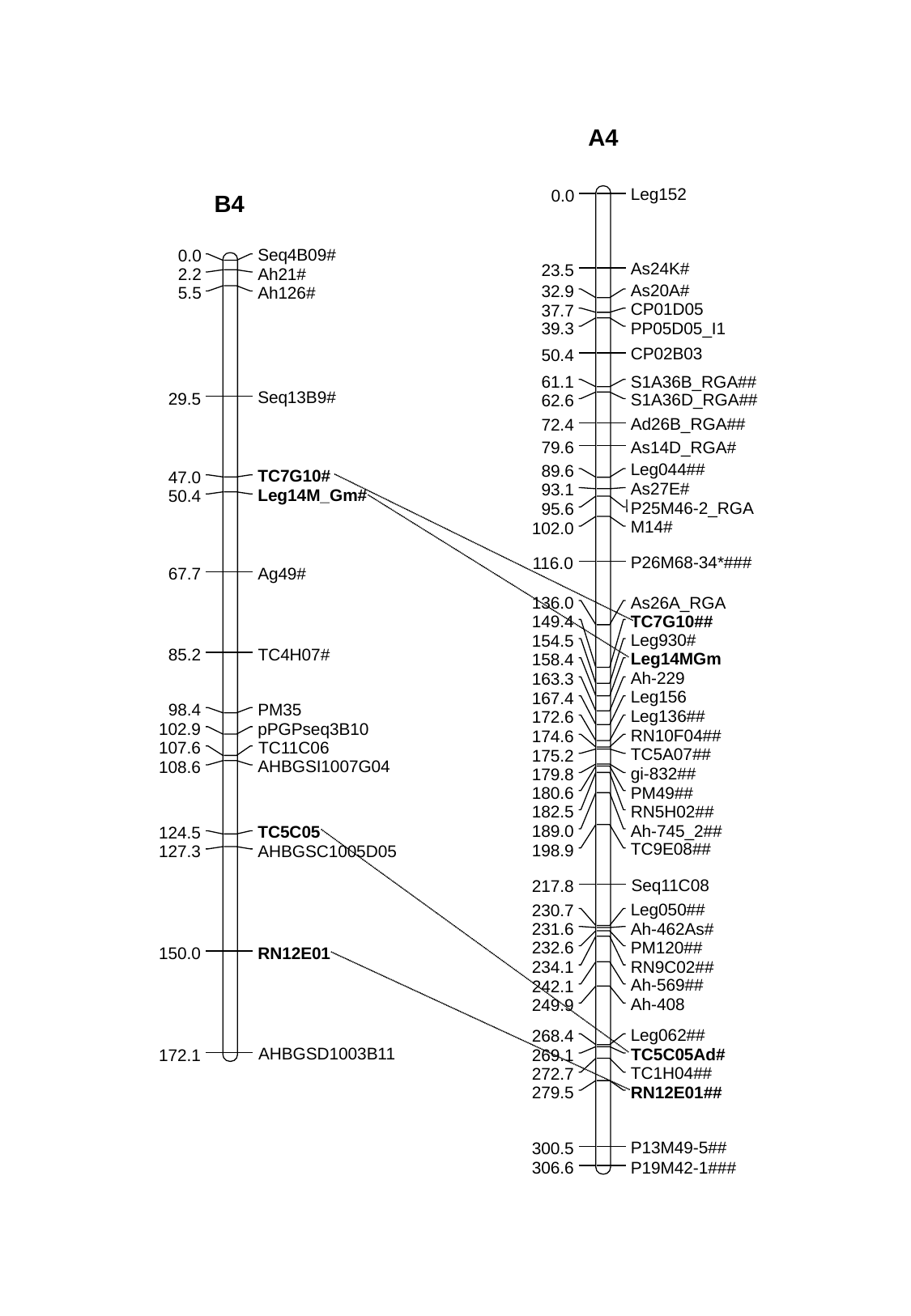

A4
Leg152
0.0
As24K#
23.5
As20A#
32.9
CP01D05
37.7
39.3
PP05D05_I1
CP02B03
50.4
61.1
S1A36B_RGA##
S1A36D_RGA##
62.6
Ad26B_RGA##
72.4
79.6
As14D_RGA#
Leg044##
89.6
As27E#
93.1
P25M46-2_RGA
95.6
M14#
102.0
P26M68-34*###
116.0
136.0
As26A_RGA
149.4
TC7G10##
Leg930#
154.5
Leg14MGm
158.4
Ah-229
163.3
Leg156
167.4
Leg136##
172.6
RN10F04##
174.6
TC5A07##
175.2
gi-832##
179.8
180.6
PM49##
182.5
RN5H02##
189.0
Ah-745_2##
TC9E08##
198.9
Seq11C08
217.8
Leg050##
230.7
231.6
Ah-462As#
232.6
PM120##
234.1
RN9C02##
Ah-569##
242.1
Ah-408
249.9
Leg062##
268.4
TC5C05Ad#
269.1
TC1H04##
272.7
279.5
RN12E01##
P13M49-5##
300.5
306.6
P19M42-1###
B4
Seq4B09#
0.0
2.2
Ah21#
5.5
Ah126#
Seq13B9#
29.5
TC7G10#
47.0
Leg14M_Gm#
50.4
67.7
Ag49#
85.2
TC4H07#
98.4
PM35
102.9
pPGPseq3B10
107.6
TC11C06
AHBGSI1007G04
108.6
TC5C05
124.5
127.3
AHBGSC1005D05
150.0
RN12E01
AHBGSD1003B11
172.1

## Slide 5
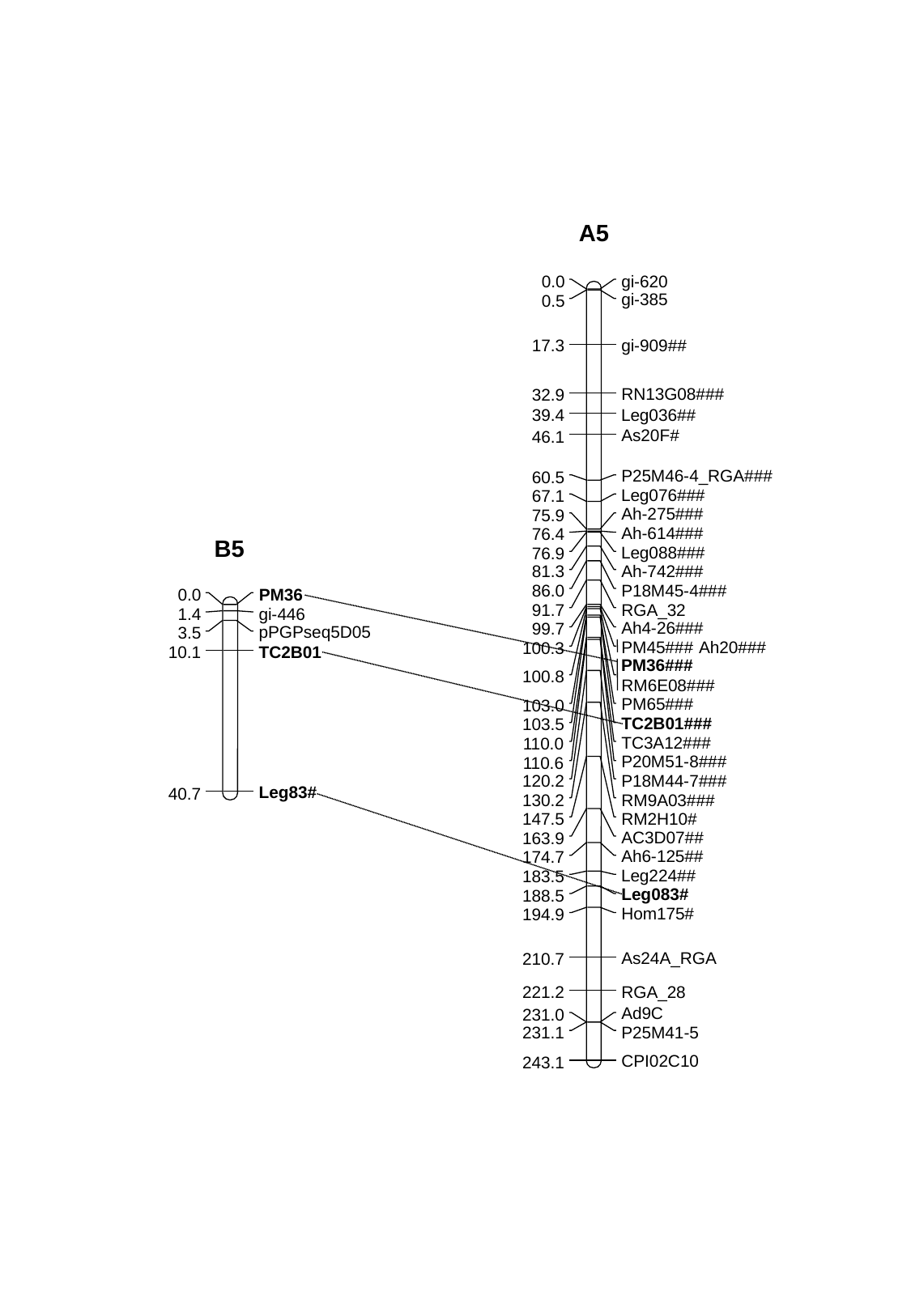

A5
0.0
gi-620
gi-385
0.5
17.3
gi-909##
RN13G08###
32.9
39.4
Leg036##
As20F#
46.1
P25M46-4_RGA###
60.5
Leg076###
67.1
Ah-275###
75.9
Ah-614###
76.4
Leg088###
76.9
81.3
Ah-742###
86.0
P18M45-4###
91.7
RGA_32
Ah4-26###
99.7
PM45###
Ah20###
100.3
PM36###
100.8
RM6E08###
PM65###
103.0
TC2B01###
103.5
TC3A12###
110.0
P20M51-8###
110.6
120.2
P18M44-7###
130.2
RM9A03###
147.5
RM2H10#
AC3D07##
163.9
Ah6-125##
174.7
Leg224##
183.5
Leg083#
188.5
Hom175#
194.9
As24A_RGA
210.7
221.2
RGA_28
Ad9C
231.0
231.1
P25M41-5
CPI02C10
243.1
B5
0.0
PM36
1.4
gi-446
pPGPseq5D05
3.5
10.1
TC2B01
Leg83#
40.7

## Slide 6
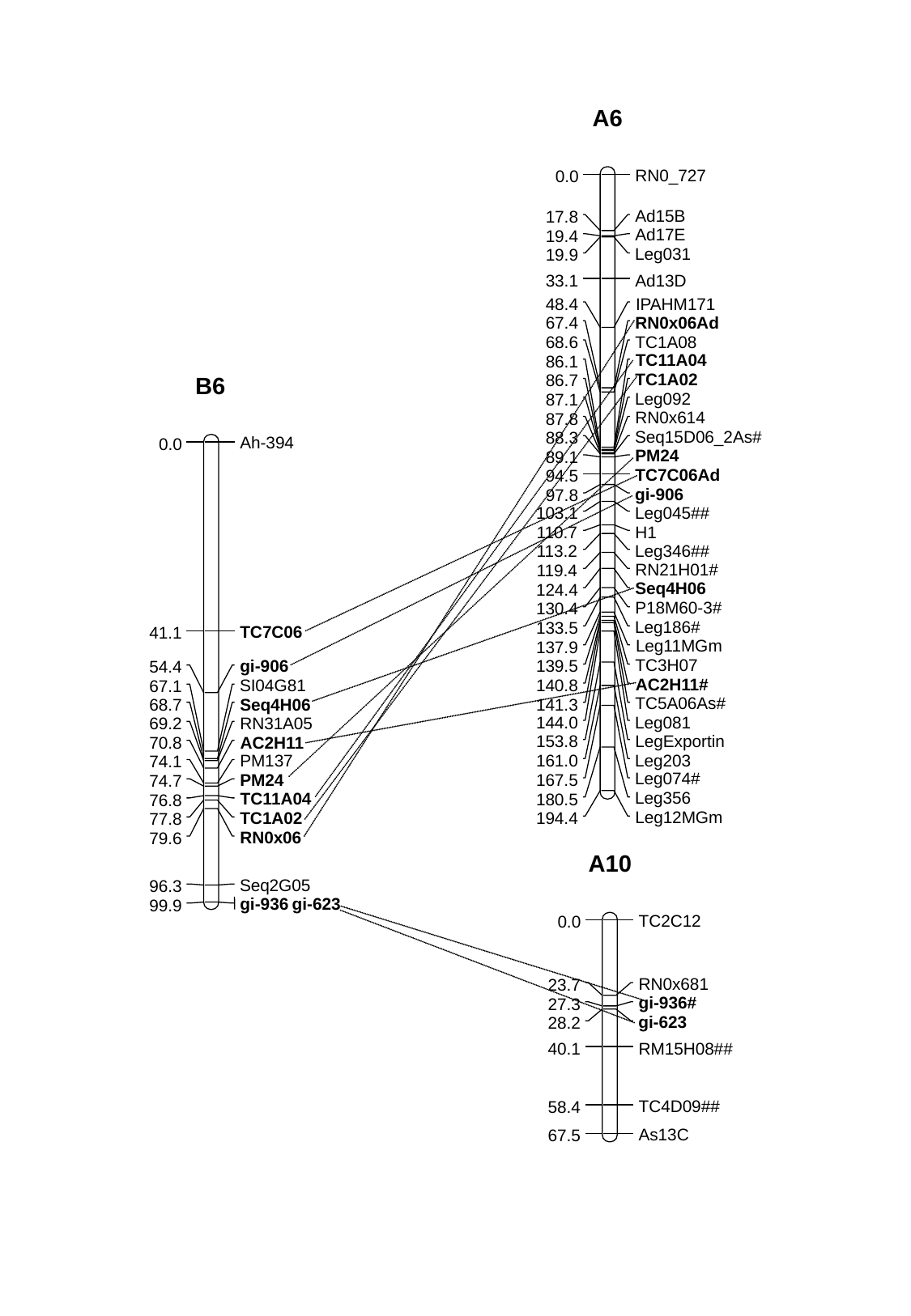

A6
RN0_727
0.0
Ad15B
17.8
Ad17E
19.4
Leg031
19.9
33.1
Ad13D
48.4
IPAHM171
67.4
RN0x06Ad
68.6
TC1A08
TC11A04
86.1
TC1A02
86.7
Leg092
87.1
RN0x614
87.8
Seq15D06_2As#
88.3
PM24
89.1
TC7C06Ad
94.5
gi-906
97.8
103.1
Leg045##
110.7
H1
113.2
Leg346##
RN21H01#
119.4
Seq4H06
124.4
P18M60-3#
130.4
Leg186#
133.5
Leg11MGm
137.9
TC3H07
139.5
AC2H11#
140.8
TC5A06As#
141.3
144.0
Leg081
153.8
LegExportin
161.0
Leg203
Leg074#
167.5
Leg356
180.5
Leg12MGm
194.4
B6
Ah-394
0.0
TC7C06
41.1
gi-906
54.4
SI04G81
67.1
68.7
Seq4H06
69.2
RN31A05
70.8
AC2H11
PM137
74.1
PM24
74.7
TC11A04
76.8
TC1A02
77.8
RN0x06
79.6
Seq2G05
96.3
gi-936
gi-623
99.9
A10
TC2C12
0.0
RN0x681
23.7
gi-936#
27.3
gi-623
28.2
40.1
RM15H08##
TC4D09##
58.4
As13C
67.5

## Slide 7
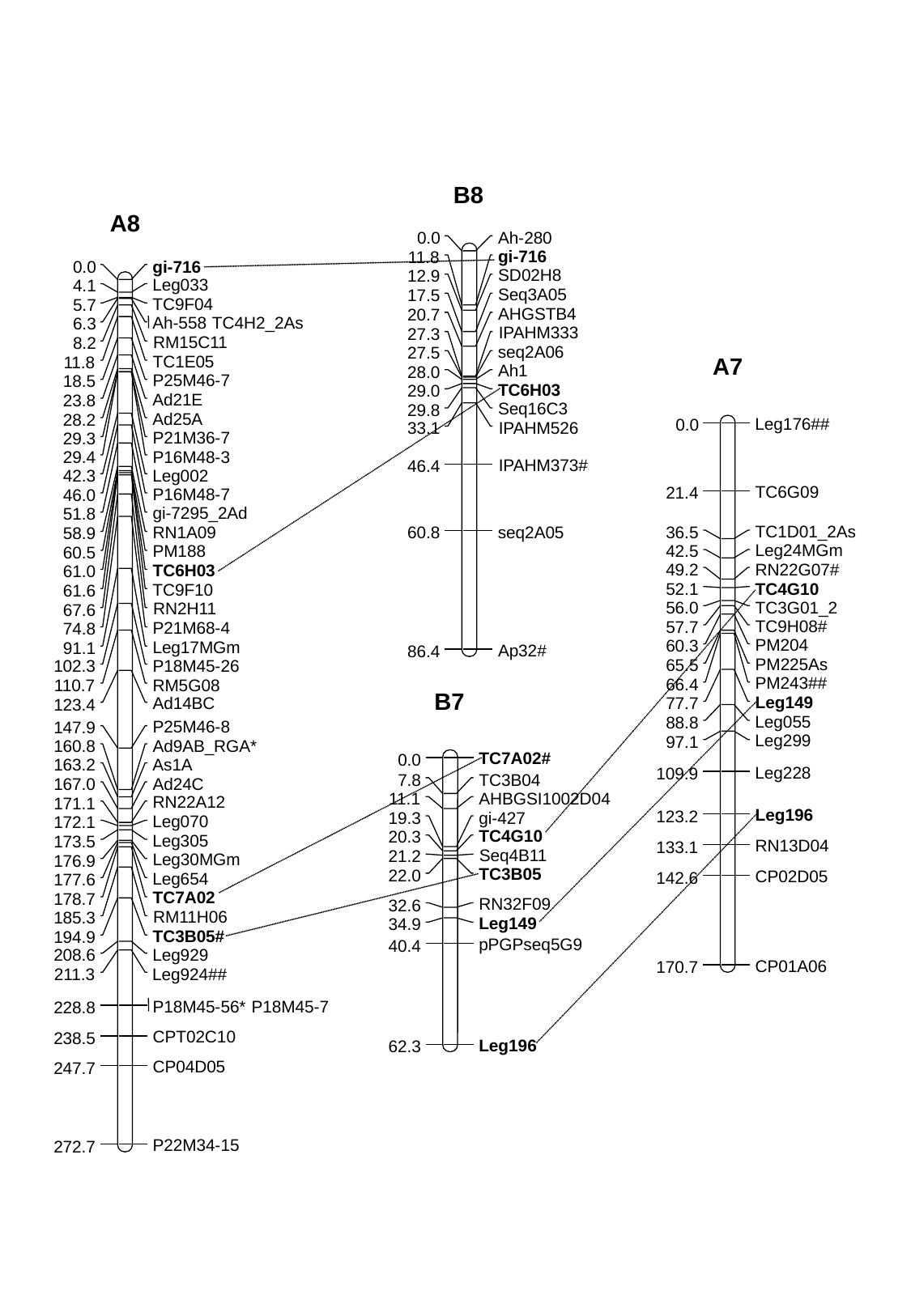

B8
0.0
Ah-280
gi-716
11.8
SD02H8
12.9
Seq3A05
17.5
AHGSTB4
20.7
IPAHM333
27.3
seq2A06
27.5
Ah1
28.0
TC6H03
29.0
Seq16C3
29.8
33.1
IPAHM526
IPAHM373#
46.4
60.8
seq2A05
Ap32#
86.4
A8
0.0
gi-716
Leg033
4.1
TC9F04
5.7
Ah-558
TC4H2_2As
6.3
RM15C11
8.2
TC1E05
11.8
P25M46-7
18.5
Ad21E
23.8
Ad25A
28.2
P21M36-7
29.3
29.4
P16M48-3
42.3
Leg002
P16M48-7
46.0
gi-7295_2Ad
51.8
RN1A09
58.9
PM188
60.5
TC6H03
61.0
TC9F10
61.6
RN2H11
67.6
P21M68-4
74.8
Leg17MGm
91.1
102.3
P18M45-26
110.7
RM5G08
Ad14BC
123.4
P25M46-8
147.9
160.8
Ad9AB_RGA*
163.2
As1A
167.0
Ad24C
RN22A12
171.1
Leg070
172.1
Leg305
173.5
Leg30MGm
176.9
Leg654
177.6
TC7A02
178.7
RM11H06
185.3
TC3B05#
194.9
208.6
Leg929
211.3
Leg924##
P18M45-56*
P18M45-7
228.8
CPT02C10
238.5
CP04D05
247.7
P22M34-15
272.7
A7
Leg176##
0.0
TC6G09
21.4
TC1D01_2As
36.5
Leg24MGm
42.5
49.2
RN22G07#
52.1
TC4G10
56.0
TC3G01_2
TC9H08#
57.7
PM204
60.3
PM225As
65.5
PM243##
66.4
Leg149
77.7
Leg055
88.8
Leg299
97.1
Leg228
109.9
Leg196
123.2
RN13D04
133.1
CP02D05
142.6
CP01A06
170.7
B7
TC7A02#
0.0
7.8
TC3B04
11.1
AHBGSI1002D04
19.3
gi-427
TC4G10
20.3
Seq4B11
21.2
TC3B05
22.0
RN32F09
32.6
Leg149
34.9
pPGPseq5G9
40.4
Leg196
62.3

## Slide 8
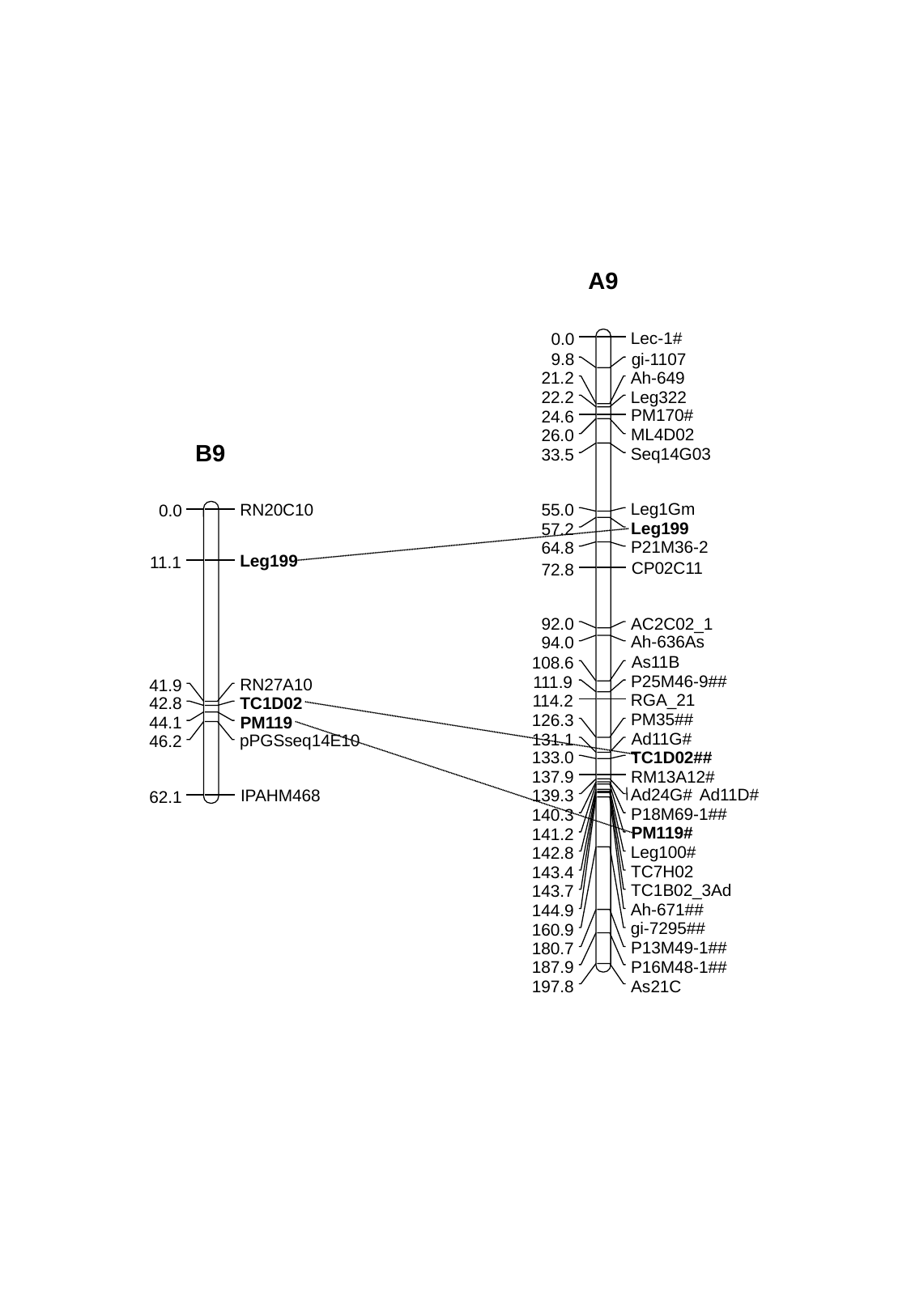

A9
Lec-1#
0.0
9.8
gi-1107
21.2
Ah-649
22.2
Leg322
PM170#
24.6
ML4D02
26.0
Seq14G03
33.5
Leg1Gm
55.0
Leg199
57.2
P21M36-2
64.8
CP02C11
72.8
92.0
AC2C02_1
Ah-636As
94.0
As11B
108.6
P25M46-9##
111.9
RGA_21
114.2
PM35##
126.3
Ad11G#
131.1
133.0
TC1D02##
137.9
RM13A12#
Ad24G#
Ad11D#
139.3
P18M69-1##
140.3
PM119#
141.2
Leg100#
142.8
TC7H02
143.4
TC1B02_3Ad
143.7
Ah-671##
144.9
gi-7295##
160.9
P13M49-1##
180.7
187.9
P16M48-1##
197.8
As21C
B9
RN20C10
0.0
Leg199
11.1
RN27A10
41.9
42.8
TC1D02
44.1
PM119
pPGSseq14E10
46.2
IPAHM468
62.1
